# Moderating Effects of Physical Activity on the Relationship between Adverse Childhood Experiences and Health-Related Quality of Life

**DOI:** 10.3390/ijerph19020668

**Published:** 2022-01-07

**Authors:** Ingyu Moon, Junghee Han

**Affiliations:** 1 School of Social Work, Nyack College, New York, NY 10004, USA; 2 Department of Social Work, University of Southern Indiana, Evansville, IN 47712, USA; jhan2@usi.edu

**Keywords:** adverse childhood experiences, physical activity, health-related quality of life, childhood abuse, household dysfunction, mental health, physical health

## Abstract

The effects of adverse childhood experiences (ACEs) on health-related quality of life (HRQOL) and their associations with physical activities (PA) are well-documented. However, the specific effects of different types of ACEs (e.g., child abuse and household dysfunction) on HRQOL and the role of PA are inconclusive. The purpose of this study is to find the buffering role of PA as a moderator that may reduce the negative impact of ACEs in general and the specific effects of different types of ACEs on HRQOL, perceived physical health, and mental health over time. The 2019 Behavioral Risk Fact Surveillance System (BRFSS), a state-based surveillance system supported by the CDC in the U.S., was used for this study. A total of 127,370 respondents from 17 states were selected for this study. First, descriptive statistics were generated and correlation analyses were conducted to find the association among variables and examine the possible predictors of HRQOL. Moderation models were then tested using Structural Equation Modeling (SEM). HRQOL in adults is negatively associated with ACEs, but is positively associated with PA. We found buffering effects of physical activity in the following relationships: (1) child abuse and HRQOL, (2) child abuse and perceived physical health, (3) ACEs and perceived mental health, (4) child abuse and perceived mental health, and (5) household dysfunction and perceived mental health. Our findings suggest that improvement of PA level is a significant predictor of improved HRQOL of adults with ACEs.

## 1. Introduction

Adverse childhood experiences (ACEs) are a set of childhood traumatic events, and their association with a panoply of risks have become the focus of numerous studies during the past two decades [1,2,3]. ACEs occur during childhood or adolescence, but they have lifelong effects that manifest in adulthood [4]. For example, in the 2016–2017 National Survey of Children’s Health, approximately half of children aged between 6 and 17 years in the U.S. reported at least one ACE [5]. Similar results were observed in the 2011–2014 Behavioral Health Risk Factor Surveillance System. Overall, 23.53% of respondents reported at least one ACE, 13.38% reported two ACEs, and 24.64% reported three or more ACEs [6]. While there are ten types of ACEs, one of the most extensive investigations of these experiences is conducted by the Centers for Disease Control and Prevention (CDC) and the Kaiser Permanente ACE study and focuses mainly on three categories: abuse (i.e., emotional, physical, and sexual abuse), household challenges (intimate partner violence (IPV), parental separation, incarcerated family member, substance abuse, and mental illness in the household), and neglect (emotional and physical neglect) [7]. Past results have shown that the most common ACE domain was emotional abuse (33.5%), followed by parental separation/divorce (28.2%), household substance abuse (26.8%), IPV (17.8%), physical abuse (17.5%), household mental illness (16.2%), sexual abuse (11.3%), and an incarcerated household member (8.1%) [8] (p. 4).

A variety of social, environmental, and structural conditions are associated with the risks of exposure to childhood adversity [9], for example, variations among socio-demographic groups. Previous research has shown that women, younger adults, those with a low level of education, those with a low income, and multi-racial study participants were more likely to report higher ACEs than their counterparts [6]. Moreover, ACEs can lead to adverse outcomes later in life. For example, ACEs can affect critical social outcomes (e.g., school-related issues, academic failure, and difficulties with socio-emotional maladjustment) [10,11,12] and health outcomes (e.g., psychosocial well-being later in life, increased morbidity, and pre-mature mortality) [13,14,15,16,17,18]. It is also worth noting that poor health-risk behaviors (e.g., substance use, violence, smoking, alcohol use, an unhealthy diet, sleep disturbance, and a lack of physical activity) are commonly observed among adults who had ACEs [19]. Exposure to ACEs causes long-term damage to the nervous system, endocrine system, immune system, and cognitive development, leading to the adoption of poor health behaviors and health outcomes in adulthood [20,21].

Recent studies have highlighted the negative relationship between ACEs and health-related quality of life (HRQOL), which is a multidimensional concept that includes physical, mental, emotional, and social functioning and the perceived quality of life of an individual [22,23]. According to the CDC Healthy Days measures, the HRQOL is also “defined as perceived physical and mental health over time” [24] (p. 1). Therefore, HRQOL is a valuable indicator of perceived physical and mental health [25,26]. A previous study that used the CDC’s healthy days measure revealed that an ACE was associated with poor HRQOL and indicated greater than 14 unhealthy days [21]. However, the authors of a recent study argued that HRQOL is a valuable measure for addressing health disparities and designing population-level interventions [27]. It is also essential to study the association between ACEs and HRQOL to develop and evaluate effective interventions for increased health outcomes among individuals with ACEs.

**Figure 1:**
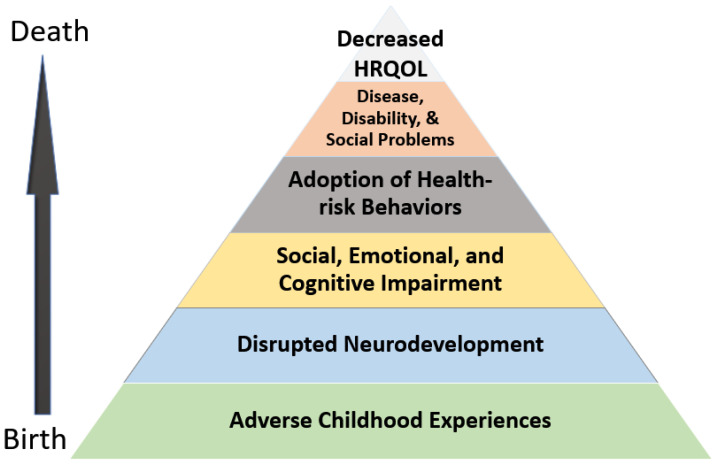
ACE Pyramid for HRQROL—modified according to potential influences throughout the lifespan of ACE [28].

According to the ACE Pyramid, which was developed by the CDC-Kaiser Permanente ACE study and outlines the impact of ACEs, these experiences are closely associated with the development of risk factors for health outcomes (see Figure 1). These outcomes include social, emotional, and cognitive impairment, diseases, the adoption of health-risk behaviors, poor health outcomes, and early death. Our modified ACE pyramid shows the causal chain of the potential influence of ACEs on the health outcomes concerning the HRQOL. As shown in the ACE pyramid, the disruption of the body’s stress response system due to ACEs can be coupled with the adoption of health-risk behaviors to cope with stress, which in turn contribute to the deterioration of health in adults.

One health-risk behavior that has been the focus of recent research is physical activity (see Figure 1). Numerous studies found that regular moderate physical activity (PA) decreased the risks of chronic diseases such as cardiovascular disease, diabetes, and cancer [29,30,31]. Additionally, it is well-known that exercise is an excellent way for individuals to manage psychological distress and ease feelings of anxiety and depression [32,33]. As expected, participation in PA has been shown to significantly benefit individuals who have had ACEs by increasing their self-esteem and reducing anxiety and depression [34]. A recent study revealed that PA has a buffering effect and reduces the impact of ACEs on depressive symptoms as well as negates the effect of depression on functional dependence in activities of daily living (ADLs) [35].

**Figure 2:**
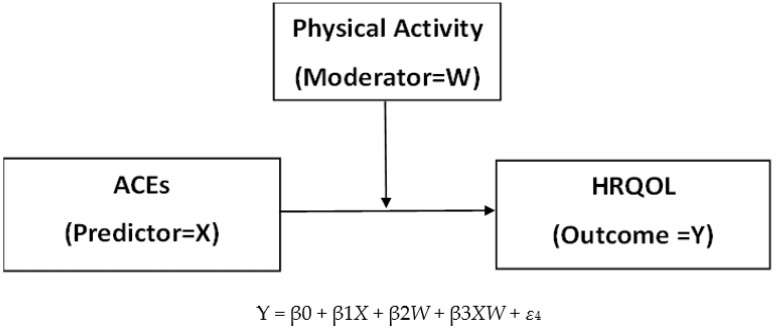
Framework of the moderation model—the role of PA in the relationship between ACE and HRQOL. Note: This is a basic model. Models with sub-domains of ACEs and HRQOL are omitted.

Based on previous findings and the ACE Pyramid, a framework of the moderation model—the role of PA in the relationship between ACEs and HRQOL—was developed in the present study (see Figure 2). We hypothesized that PA plays a moderating role between ACEs and HRQOL. However, the specific effects of certain types of ACEs (i.e., child abuse and household dysfunction) on HRQOL and the moderating role of PA are unclear. More importantly, the specific effects of certain types of ACEs on the HRQOL sub-domains (perceived physical health and mental health) have not been sufficiently explored. It is important to note that the sub-domains of ACEs and childhood maltreatment and dysfunction have a relatively unequal impact on mental health [36]. Therefore, this study aimed to examine the possible moderating role of PA in reducing the negative impact of ACEs and the specific effects of various types of ACEs (i.e., child abuse and household dysfunction) on HRQOL and perceived physical and mental health. The research questions addressed in the study are as follows:

(1) Do ACEs and the sub-domains (i.e., child abuse and household dysfunction) negatively influence HRQOL, physical health over time, and mental health over time, even when numerous covariates are controlled for?
(2) Does the level of PA moderate the effect of ACEs and the sub-domains (i.e., child abuse and household dysfunction) on HRQOL, physical health over time, and mental health over time, even when numerous covariates are controlled for?

## 2. Materials and Methods

### 2.1. Study Sample

This study utilized the 2019 Behavioral Risk Fact Surveillance System (BRFSS), which is a state-based surveillance system that is supported by the CDC in the U.S. The BRFSS is a cross-sectional telephone survey that involves the accumulation of data on health-related behaviors, the use of preventive services, chronic conditions, and health status [37]. Trained interviewers for the BRFSS collect data for each calendar month by conducting landline and cellular telephone surveys in 50 states of the U.S., the District of Columbia, Guam, and Puerto Rico. In 2019, 17 states included questions on ACEs, an optional module of the BRFSS. A total of 127,370 respondents from the 17 states that used the ACE module were selected for this study. All the individuals who participated in the 2019 BRFSS were noninstitutionalized adults who were aged 18 years or older.

The demographic information (*n* = 127,370) is listed in Table 1. Since the data were gathered via a complex survey design, the weighted % (proportion) was used for descriptive analysis. Most participants were 65 years or older (23.4%) and were of White, non-Hispanic (70.5%) ethnicity. There was no gender difference among the participants. Greater than half of the participants attended college or graduated from college and were employed for a wage or were self-employed. A total of 21.1% of the respondents were retired. Approximately 54% of the respondents were married or lived with a significant other. Regarding health-related factors, greater than 90% of the respondents had health insurance and approximately two-thirds of respondents (60.8%) had at least one chronic disease such as diabetes, asthma, COPD, cancer, angina, heart attack, kidney disease, stroke, or depression. Additionally, 69.5% of the respondents reported that they were overweight or obese.

### 2.2. Measures

According to the CDC, the BRFSS questionnaire is composed of an annual standard core, optional modules, and state-added questions [37]. HRQOL, PA, and socio-demographic variables are included in the core module, while ACEs are listed in the optional modules. In 2019, a total of 17 states chose the optional ACE module. Several variables (e.g., level of PA, age, race, income, etc.) were calculated from responses provided during phone interviews. Most measures in the BRFSS showed high reliability and validity [38].

1. HRQOL, Mentally Unhealthy Days, and Physically Unhealthy Days: Dependent Variables. The CDC suggests a summary index of “unhealthy days” (a maximum of 30 mentally and physically unhealthy days) as a measurement to assess HRQOL [39]. The unhealthy days index includes the following questions: (a) Now thinking about your physical health, which includes physical illness and injury, for how many days during the past 30 days was your physical health not good? and (b) Now thinking about your mental health, which includes stress, depression, and problems with emotions, for how many days during the past 30 days was your mental health not good? These two questions are then combined to calculate a summary index of the overall unhealthy days, with a logical maximum of 30 unhealthy days. The unhealthy days index assumes an independent relationship between the two types of days [40]. In the present study, the unhealthy days were converted into HRQOL-healthy days (the positive complementary form of unhealthy days) by subtracting the number of unhealthy days from 30. Therefore, the HRQOL range for this study was 0–30. The BRFSS HRQOL measure has also shown acceptable test–retest reliability and strong internal validity [24]. In addition to HRQOL-healthy days, physically unhealthy days and mentally unhealthy days were also used in this study as dependent variables to examine the difference between the impact of the independent variables and the moderating variables on mental and physical health. Therefore, three continuous dependent variables were used in this study (range 0–30). For the descriptive statistics, poor physical health was assigned to individuals who reported having 14 or more days of poor physical health during the previous 30 days. Poor mental health was assigned to individuals who reported having 14 or more poor mental health days during the previous 30 days [41].

2. ACEs, Child Abuse, and Household Dysfunction: Independent Variables. The ACE module in the BRFSS consists of 11 questions that refer to a respondent’s first 18 years of life. These 11 questions are categorized into eight domains: physical abuse, emotional abuse, sexual abuse, household mental illness, household substance use, household domestic violence, incarcerated household member, and parental separation or divorce [6]. These eight domains are also divided into two sub-domains: child abuse (i.e., emotional abuse, physical abuse, and sexual abuse) and household dysfunction (i.e., household mental illness, household substance use, household domestic violence, parental separation or divorce, and an incarcerated household member). For this research, three variables were utilized: child abuse (range 0–3), household dysfunction (range 0–5), and ACEs (range 0–8), and higher scores indicated greater exposure to ACEs. In terms of psychometrics, the overall internal consistency for the BRFSS ACE module is reported to be 0.78, with subscale alphas ranging from 0.61 to 0.80. For descriptive purposes, three main ACE score groups were used in this study: “0 ACEs”, “1–2 ACEs”, and “3+ ACEs”. This grouping is compatible with the scoring recorded in the reports of several states on ACEs [39,42].

3. PA: The Moderator Variable. The level of PA was assessed subjectively using questions from the BRFSS that described four PA levels, as follows: highly active, active, insufficiently active, and inactive. The study participants reported the quantity, frequency, and intensity of PA. From these self-reports, the level of PA in minutes per week was calculated as a variable for the PA categories. The participants in the “highly active” category performed enough PA per week to meet the 300-minute (or the vigorous equivalent) aerobic activity recommendation. The participants who reported that they performed 150–300 minutes per week (or the vigorous equivalent) of PA were labeled as “active” respondents. The participants who engaged in insufficient PA (11–149 minutes per week) were described as “insufficiently active” for their level of PA, and those who reported no PA were termed “inactive.” This categorization is in accordance with the levels of PA recommended by Healthy People 2010, and the BRFSS PA measurement has a fair validity and test–retest reliability [43,44,45].

4. Socio-Demographic and Health Variables: Covariates. This study included various socio-demographic characteristics and health factors as covariates. These included age (six age groups in order), sex (female vs. male), race (White vs. non-White), income (seven income levels), educational attainment (three categories), marital status (married and living with a significant other vs. single), obesity (BMI ≥ 25 vs. BMI < 25), the existence of chronic disease, and health care coverage. These variables were reported as contributing factors to HRQOL in the literature [26,46,47,48].

### 2.3. Data Analysis

The BRFSS is a large-scale, federally funded survey that uses a complex, multistage, probability sampling design [49]. Since the data are collected using a complex survey design, data weighting helps to make the data more representative of the population from which the data was collected. Therefore, in the present study, weights were assigned to the data during analysis and the conducting of statistical tests. Moreover, Stata 13 was used in this study [50]. To test the relationship between ACEs (child abuse and household dysfunction) and HRQOL as moderated by the levels of PA, we selected moderation analyses. Thus, the examination of the interaction effect of ACEs and PA allowed us to explore how PA may weaken the relationship between the independent and dependent variables [51].

Descriptive statistics were generated and correlation analyses were conducted to investigate the associations among the variables and examine the possible predictors of HRQOL. Prior to running the moderation analysis, preliminary analyses were performed, including multivariate normality, multicollinearity, and missingness. The moderation models were then tested using structural equation modeling (SEM). The moderation analysis included multiple steps. First, mean-centering was applied to the independent variables (X1, X2, and X3) and moderator (W) to minimize the chance of multicollinearity [52]. Second, the interaction term of the independent variables and a moderating variable were created (i.e., X1 × W, X2 × W, and X3 × W) and were included in the moderation models. Next, the interaction effects between the independent variables (ACEs, child abuse, and household dysfunction) and the moderator variable (PA) on the dependent variables were examined through SEM [53]. To delineate the interaction effects of PA and ACEs on HRQOL, the effects of ACEs on HRQOL at low and high values of PA were also plotted.

## 3. Results

Table 2 shows the baseline measures of the key variables. Concerning the dependent variable, HRQOL, 13.5% of respondents reported poor physical health, and 14.4% reported poor mental health for the previous 30 days. On average, the respondents reported 22.5 healthy days out of 30 days for physical and mental health. Specifically, the respondents experienced an average of 4.3 days of physically unhealthy days and 4.6 days of mentally unhealthy days. Similar results were observed for the physically and mentally healthy days (25.7 and 25.4 days, respectively). The prevalence of ACEs score among the 17 states showed that many of the respondents experienced at least one ACE (36.5%) and approximately one-fourth (26.1%) of the respondents experienced three or more ACEs. Approximately 40% of the respondents experienced child abuse, and greater than half (52.8%) of the respondents experienced household dysfunction. Concerning the moderator of this study, i.e., the level of PA, half of the respondents (50.8%) were in the insufficiently inactive or inactive categories.

A correlation table for the survey data was created based on the variance estimates for the correlations [54]. The bivariate correlations showed that the HRQOL was negatively correlated with the physically and mentally unhealthy days, ACEs, child abuse, and household dysfunction, and was positively correlated with age and income (Table 3). Conversely, the physically unhealthy days during the previous 30 days were positively correlated with ACEs and their subcategories and were negatively correlated with PA and income. Similar results were observed for the mentally unhealthy days, except for age. The ACEs and subcategories were negatively correlated with the level of PA.

Prior to the moderation analysis, multivariate normality, multicollinearity, and missing values were tested. First, we conducted Mardia’s coefficient to assess multivariate normality (full-joint normality) with the key variables. The application of Mardia’s test of normality to the key factors of interest (HRQOL, ACEs, and PA) revealed that the assumption of joint normality was violated because the results showed skewness (χ^2^ = 54,186.97, *p* < 0.000), and kurtosis (χ^2^ = 152.0.42, *p* = 0.000). Mardia’s tests that were conducted using the two remaining dependent variables showed similar results. Regarding multicollinearity, we observed that the VIF was not greater than 10, and the tolerance value was not less than 0.1, which are acceptable values [55]. To investigate an assumption for SEM, whether missing completely at random (MCAR) occurred, Little’s MCAR test and covariate-dependent missingness (CDM) were performed. The result of Little’s MCAR test for the key variables provided an χ^2^ distance of 14,578.7 (*df* = 6509; *p* < 0.001), indicating that the missing data were not MCAR at a significance level of *p* ≥ 0.001 [56]. However, the result of CDM was not significant (χ^2^ distance = 16,401.2; *df* = 47,762; *p* > 0.05). This implies that the missing data mechanisms can be reasonably viewed as CDM, which is a special case of missing at random because the patterns of missing values depend on the possible covariates [56]. To reduce the effect of non-normality and missing values, we used a maximum likelihood (ML) estimation, which is the variance–covariance matrix of the estimators and listwise deletion [57]. Even with a nonnormal distribution curve, the ML parameter estimates involved less bias and were more efficient with medium (over 120) to large samples [58]. Thus, the assumption of multivariate normality (full-joint normality) of the key variables may be relaxed in SEM when using ML.

The results of the moderation analysis are shown in Table 4. Nine models were tested for this study. According to the model fit tests, the coefficient of determination (=R^2^ in SEM) indicated that the models were not a perfect fit; the CD ranged from 0.136 to 0.186 in the nine models. However, the standardized root mean square residual (SRMR) was 0.000, which is a recommended value. In all the models, the covariates of race, the existence of chronic disease and the employment status, marital status, and income were significant predictors of HRQOL, perceived physical health, and mental health. The covariates of gender and health coverage were not significant in models 4, 5, and 6, and educational attainment was not significant in models 1, 7, 8, and 9. HRQOL was used in the first three models as a dependent variable to examine the effect of ACEs and the subcategories, including child abuse and household dysfunction. Physically unhealthy days were used as a dependent variable in models 4–6, and mentally unhealthy days were used as a dependent variable in models 7–9. Of the three models that included HRQOL, only the interaction between the level of child abuse and PA revealed statistical significance (*B* = 0.15; *t* = 2.39; *p* = 0.017; 95% CI: 0.03 to 0.27). Interaction effects regarding ACE * PA and household dysfunction*PA were nonexistent. The effect of ACEs and household dysfunction on physically unhealthy days were not moderated by PA, while the effect of child abuse on physically unhealthy days decreased according to the level of PA (*B* = −0.13; *t* = −0.2.51; *p* = 0.014; 95% CI: −0.23 to −0.07). The interactions between ACEs * PA (*B* = −0.09; *t* = −3.35; *p* = 0.001; 95% CI: −0.14 to −0.03), child abuse * PA (*B* = −0.21; *t* = −3.70; *p* = 0.000; 95% CI: −0.32 to −0.10), and household dysfunction * PA (*B* = −0.13; *t* = −3.39; *p* = 0.001; 95% CI: −0.21 to −0.05) were proven as statistically significant predictors of mentally unhealthy days. In summary, all the models concerning mentally unhealthy days showed that PA buffered the effects of ACEs, child abuse, and household dysfunction for those days.

**Figure 3:**
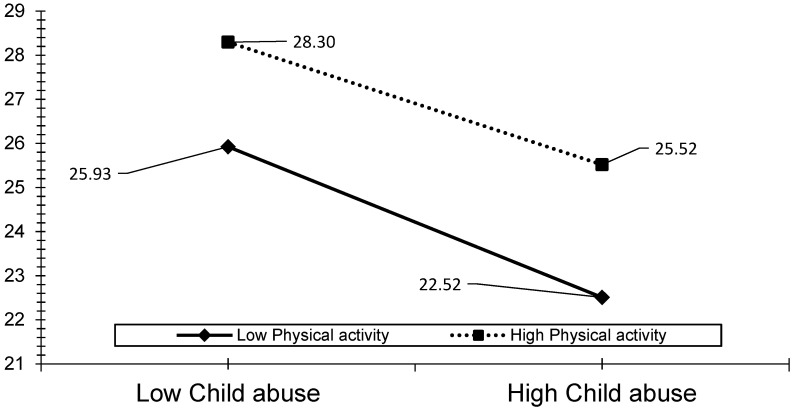
Model 2—Graph of interaction effect between PA and child abuse on HRQOL.

**Figure 4:**
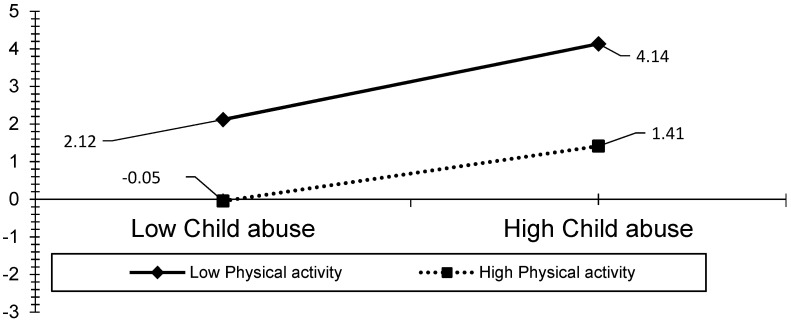
Model 5—Graph of interaction effect between PA and child abuse on physically unhealthy days.

**Figure 5:**
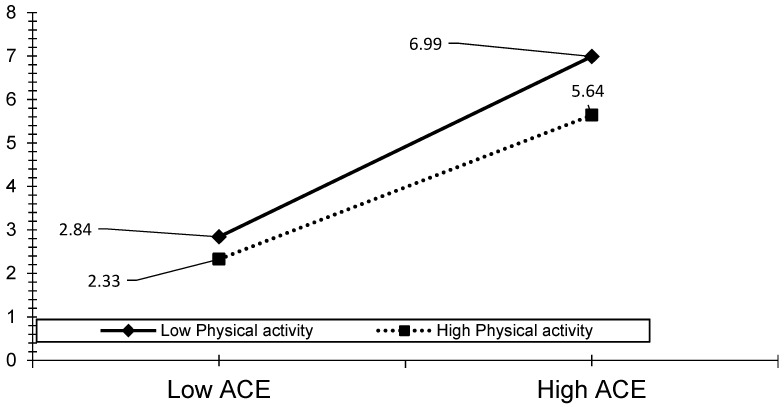
Model 7—Graph of interaction effect between PA and ACE on mentally unhealthy days.

**Figure 6:**
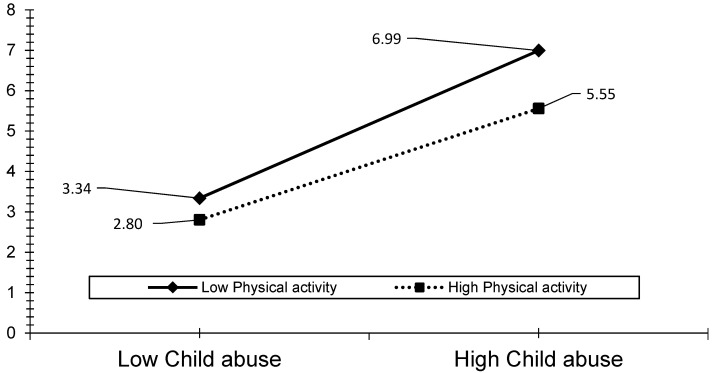
Model 8—Graph of interaction effect between PA and child abuse on mentally unhealthy days.

**Figure 7:**
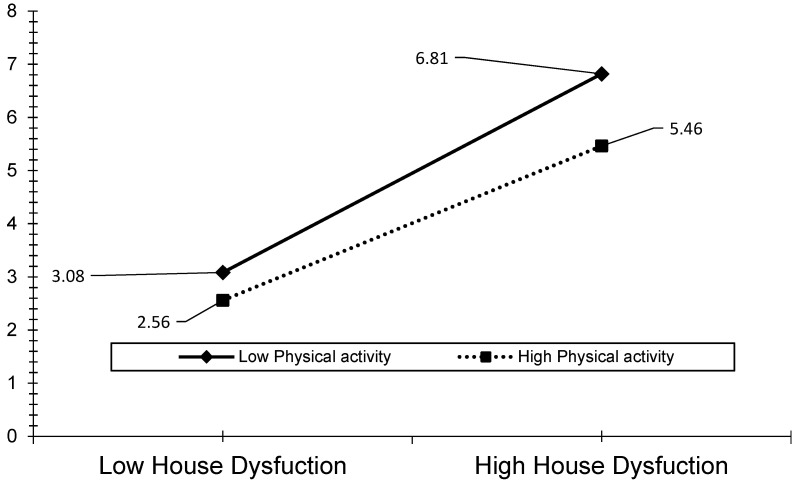
Model 9—Graph of interaction effect between PA and household dysfunction on mentally unhealthy days.

The visual analyses also revealed that the effect of ACEs, child abuse, and household dysfunction could be reduced by the level of PA (see Figure 3, Figure 4, Figure 5, Figure 6 and Figure 7). The results presented in Figure 3 show that individuals who performed a high level of PA showed higher HRQOL even if they had experienced a higher level of child abuse. The results presented in Figure 4 show that individuals who performed a high level of PA reported fewer days of being physically unhealthy. This suggests that increased PA is associated with a decrease in physically unhealthy days and minimizes the effect of child abuse on physically unhealthy days. The results presented in Figure 5, Figure 6 and Figure 7 show that the relationships between all three independent variables and mentally unhealthy days were weaker as the level of PA increased. The steeper the slope of PA, the greater the magnitude of the impact of the ACEs and subcategories on the HRQOL, physically unhealthy days, and mentally unhealthy days.

## 4. Discussion

Most of our findings were consistent with our hypotheses. The ACEs and sub-domains were negatively associated with the HRQOL, while they were positively associated with physically unhealthy days and mentally unhealthy days over time. These results indicate that greater exposure to ACEs, including child abuse and household dysfunction, corresponds to decreased HRQOL when various socio-demographic and health variables are controlled for. These results are consistent with those reported in previous studies, in which a relationship between ACEs and decreased HRQOL in adulthood was observed [59,60]. Overall, the respondents showed a 1.45-day decrease in HRQOL per one ACE score, a 2.79-day decrease per one child abuse score, and a 1.95-day decrease per one household dysfunction score in the simple regression analysis. Similarly, relationships were observed between all three independent variables and physically and mentally unhealthy days. Moreover, our results suggest that child abuse is a stronger predictor of physically unhealthy days (a 1.18 unhealthy day increase per one score) and mentally unhealthy days (a 0.65 unhealthy day increase per one score). Therefore, it can be assumed that the experience of child abuse causes greater damage to an individual’s health compared to the experience of household dysfunction in childhood. This result is congruent with the findings of previous research, which demonstrated that childhood maltreatment is an improved predictor of poor mental health, while certain aspects of household dysfunction are not a strong predictor of mental health among older adolescents [36].

The level of PA was positively associated with the HRQOL and negatively associated with physically unhealthy days and mentally unhealthy days. The more active an individual was, the higher their reported HRQOL (i.e., healthy days). These findings corroborate those reported in previous research, which showed that physically active individuals were more likely to report greater HRQOL than those who were physically inactive [61,62,63]. It is important to note that PA is associated with elevated mental health and well-being, which are closely linked to HRQOL. Despite inconsistent results in previous studies, a PA intervention group showed a positive effect on the critical domain of HRQOL, including physical functioning, bodily pain, general health, and mental health in women compared with the control group [64]. Additionally, a recent randomized trial of an exercise program demonstrated that the participants of PA intervention programs showed improved cardiovascular health and an increase in certain areas of HRQOL such as bodily pain, daily activities, and the physical component summary [65]. The present study did not include the multidimensional sub-domains of HRQOL such as physical functioning, daily activities, and bodily pain, and focused only on physically unhealthy days and mentally unhealthy days over time. Notwithstanding this limitation, the findings of this study indicate the potential benefits of generating PA interventions for the general population because PA may reduce physically and mentally unhealthy days in adults in the U.S.

Our prediction that PA would moderate the effects of ACEs, child abuse, and household dysfunction on HRQOL was partially supported. The results revealed that as the level of PA increased, the associations between the ACEs and HRQOL became weaker. However, the moderation effect of PA between the ACEs and HRQOL was not statistically significant. In other words, the participants who reported a higher ACE score had lower levels of HRQOL and perceived physical health over time; their HRQOL and perceived physical health scores did not change significantly regardless of the activity level. Similarly, PA had little effect on the relationship between household dysfunction and perceived physical health. As shown in the results, PA moderated the effects of child abuse on HRQOL, perceived physical health, and mental health. Additionally, PA showed a buffering effect on the association between the exposure to ACEs, child abuse, and household dysfunction and perceived mental health over time. These findings are congruent with those reported in previous studies, in which PA was shown to negate the negative impact of ACEs on health outcomes, including depression and functional dependence [35]. In summary, childhood trauma is the strongest predictor of HRQOL as compared with household dysfunction, and PA can buffer the negative impact of ACEs (both child abuse and household dysfunction) on the perceived mental health of individuals. Thus, PA programs can be an effective intervention strategy regarding the physical and mental health of adults who have experienced child abuse. Additionally, PA can help to improve the mental health of individuals who have been exposed to ACEs.

PA promotes resilience and reduces levels of psychological distress [66]. However, despite the physical and psychological benefits of PA, individuals who have had ACEs and have an increased risk of developing mental disorders and other physical health issues in adulthood are less likely to be physically active (less than 90 minutes of PA per week) [67]. The authors of previous studies have proposed PA as an intervention strategy for the treatment of post-traumatic stress disorder (PTSD) symptoms and to improve the health outcomes of individuals who have PTSD [68]. Moreover, the authors suggested that clinicians who use PA as a PTSD intervention strategy should include patient-specific exercises in their treatment plan (e.g., a walking program, yoga, or aerobic activity). Furthermore, a narrative review of 19 extant studies revealed that aerobic exercise exerted a positive influence on PTSD symptoms, such as exposure and desensitization to internal arousal cues, enhanced cognitive function, exercise-induced neuroplasticity, the normalization of hypothalamic pituitary axis (HPA) function, and reductions in inflammatory markers [69] (p. 133). However, research in the field of PA interventions for PTSD is in its infancy [70]. Similarly, the importance of PA intervention in mental health care for adults who have had ACEs has been neglected.

In healthcare settings, several barriers may hinder service providers from focusing on PA in adults who have had ACEs, particularly those who have been exposed to child abuse. For example, mental health professionals may recognize the advantages of PA; however, barriers exist regarding the use of PA as an intervention strategy, for example, a “lack of time and reimbursement for health promotion activities, and inadequate practice capacity for healthcare providers, increased time and labor demands for administrative personnel; constrained access to participants and limited funding for researchers; and superseding commitments and inaccurate comprehension of the research protocol for patients” [71] (p. 81). Additionally, insufficient knowledge, a lack of motivation, financial issues, and a lack of support for the promotion of PA are barriers for social workers and relevant professionals [72]. It is worth noting that the importance of interventions that focus on PA for individuals who have had ACEs is inadequately understood and has been neglected by mainstream healthcare services. For this reason, we argue that healthcare professionals must be motivated to utilize PA as an alternative intervention strategy for adults who have had ACEs in order to improve the physical and mental health outcomes.

## 5. Limitations

This study has several limitations that require further investigation. For example, the ACE module was optional for each of the states, and only 17 of the states collected data on ACEs. Even with the inclusion of New Jersey, which is the most heavily urbanized state [73], the remaining 16 states do not contain large urban areas compared to states that have large cities, such as New York, California, and Texas. Second, due to the cross-sectional nature of this study, bi-directional relationships most likely existed between the moderating variable (PA) and the dependent variable (HRQOL). While the fact that the ACEs occurred in childhood confirmed that there was a temporal relationship with the other variables, it was not clear whether PA caused changes in the HRQOL or not. Although Baron and Kenny’s moderation models, with cross-sectional design studies, were introduced for the testing of causal relations based on theoretical or conceptual models, it was challenging to discover rigorous inferences concerning the causal relations and the cross-sectional design [52,74]. Therefore, our findings cannot guarantee causal associations among the variables over time. Third, the BRFSS is a telephone survey, which may have introduced a non-response bias for certain marginalized individuals who did not have a fixed telephone number due to institutionalization, hospitalization, or incarceration during the data collection period. Fourth, the BRFSS uses self-reported telephone interview questions, which may have led to recall bias, social desirability bias, and confirmation bias. Moreover, a bias between the older and younger respondents may have occurred because the younger respondents may have been more likely to recall childhood memories. Furthermore, certain respondents may have exaggerated or misreported their level of PA to reflect the socially desirable nature of participating in PA. Similarly, the mentally and physically unhealthy days are a subjective concept, which may rely on an individual’s psychological, educational, social, and environmental status. Thus, the decision regarding an individual’s ACEs, PA level, mental and physical health, and HRQOL may have been affected by his or her preconceptions, beliefs, or preferences due to the nature of the self-reported interview questions. Fifth, although our results suggest that physically active individuals have an improved HRQOL compared with those who are physically inactive, it is also possible that the HRQOL may be associated with other unmeasured lifestyle factors such as nutrition, smoking, substance use, leisure, the presence or absence of a support network, etc. Despite efforts to control the effect of the unmeasured factors and the high validity of the BRFSS’s HRQOL-healthy day measures, focusing solely on the perceived mental and physical health of individuals may not address all the aspects of HRQOL. Lastly, the high missing values observed in this study is a limitation. In applied research, the existence of missing values is a pervasive issue that may cause loss of information, reduced power, and bias in estimates [75]. Multiple imputation (MI), a widely used method for handling missing data under the missing at random (MAR) assumption, can minimize the bias in the estimates. Therefore, in the present study, the results of the MI applied data were compared with the results of a maximum likelihood (ML) estimation (shown in Table 3), which used the listwise deletion of missing values. As the results were similar, we reported the ML results.

## 6. Conclusions

Notwithstanding these limitations, the findings of this study indicate the potential for the generation of important information regarding the buffering role of PA between ACEs and health-related outcomes in adulthood. First, the provision of appropriate PA interventions for adults who have had ACEs, particularly those who have experienced childhood trauma, may improve the HRQOL of adults. Moreover, even adults who have had relatively mild ACEs (i.e., household dysfunction) may gain considerable benefits from increased levels of PA. Notably, for adults who have had ACEs and who have suffered from mental health issues, an improvement in the PA level is a significant predictor of an improved HRQOL. In summary, our findings support PA as a potentially critical factor in the HRQOL of individuals who have had ACEs. Future studies should examine whether PA can relieve severe health-related issues, such as the symptoms of mental illnesses (e.g., PTSD, schizophrenia, bipolar disorder, etc.) and physical illnesses (e.g., heart disease, cancer, etc.), following childhood trauma experiences among adults. Additionally, further research is required regarding the use of direct measures such as the diagnosis of mental illness by mental health professionals, heart rate monitoring, and accelerometry for PA levels. Lastly, PA can be used as an alternative intervention for mental health issues since it buffers the adverse impacts of ACEs on mental health. The authors of the present study strongly encourage healthcare providers to utilize PA as an intervention strategy for adults who have had ACEs.

## Figures and Tables

**Table 1:** Sample demographic information in 17 states in the 2019 BRFSS (*n* = 127,370).

Item	N (%)	Weighted % (Proportion)
Gender		
Female	70,336 (55.2)	51.5
Male	57,034 (44.8)	48.5
Age		
18–24	7458 (5.9)	11.9
25–34	12,831 (10.1)	16.4
35–44	14,201 (11.1)	15.4
45–54	18,374 (14.4)	15.8
55–64	26,047 (20.5)	17.1
65 and older	48,459 (38.0)	23.4
Race		
White, Non-Hispanic	100,019 (78.5)	70.5
Non-White	27,351 (21.5)	29.5
Education Attainment		
Did not graduate high school	9903 (7.8)	11.8
Graduated high school	36,694 (28.9)	30.7
Attended or graduated college	80,248 (63.3)	57.5
Employment Status		
Employed for wage or self-employed	59,769 (38.9)	55.8
Unemployed or unable to work	15,769 (12.6)	12.8
A homemaker or a student	9153 (7.3)	10.2
Retired	40,836 (32.5)	21.1
Marital Status		
Divorced, widowed, sepa rated, or never married	58,889 (46.58)	45.2
Married or a member of an unmarried couple	67,543 (53.42)	54.8
Healthcare Coverage		
Covered	11,5951 (91.5)	91.5
Not covered	10,787 (8.5)	8.5
Chronic Diseases		
1 or more diseases	77,426 (60.8)	51.5
No chronic disease	49,944 (39.2)	48.5
BMI		
BMI < 25 (Not overweight or obese)	35,507 (30.5)	31.9
BMI ≥ 25 (Overweight or obese)	81,011 (69.5)	68.1

**Table 2:** Univariate analysis of key variables (*n* = 127,370).

Item	N (%)		Weighted % (Proportion)
HRQOL			
Poor physical health	18,946 (15.3)		13.5
Poor mental health	16,205 (13.0)		14.4
ACE			
0 ACEs	41,729 (42.3)		37.3
1–2 ACEs	35,073 (35.6)		36.5
3+ ACEs	21,795 (22.1)		26.1
Child Abuse			
Yes	37,559 (36.9)		40.1
Household dysfunction			
Yes	48,485 (47.4)		52.8
PA Level			
Inactive	36,812 (32.5)		30.9
Insufficiently active	20,527 (18.1)		19.9
Active	18,399 (16.2)		17.2
Highly Active	37,663 (33.2)		32.0
	Mean	SE	Range: Min–Max
HRQOL (Healthy days)	22.48	0.06	0–30
Physically unhealthy days	4.30	0.44	0–30
Mentally unhealthy days	4.56	0.47	0–30

**Table 3:** Correlation matrix for continuous predictors, moderator, and outcome variables (*n* = 77,709).

	1. HRQOL	2. Physically Unhealthy Days	3. Mentally Unhealthy Days	4. ACEs	5.Child Abuse	6. Household Dysfunction	7. Physical Activity
1.	1						
2.	−0.82 ***	1					
3.	−0.73 ***	0.33 ***	1				
4.	−0.24 ***	0.13 ***	0.27 ***	1			
5.	−0.21 ***	0.12 ***	0.23 ***	0.82 ***	1		
6.	−0.21 ***	0.10 ***	0.25 ***	0.91 ***	0.53 ***	1	
7.	0.19 ***	−0.20 ***	−0.11 ***	−0.03 ***	−0.01 ***	−0.03 ***	1

*Note*: Numbers with a star indicate statistical significance. *** *p* < 0.001.

**Table 4:** Moderation analysis of PA on the relationships between comorbidity, psychological distress, and HRQOL (*n* = 77,553).

Predictor	*B*	*SE*	β	*t*	95% CI	
					Lower	Upper
Model 1 (X1 * W *→* HRQOL); *SRMR = 0.000 CD = 0.186*						
ACEs	−1.13	0.04	−0.20	−29.28 ***	−1.20	−1.05
PA	0.99	0.05	0.11	18.22 ***	0.88	1.09
ACEs * PA	0.03	0.03	0.01	1.00	−0.03	0.09
Model 2 (X2 * W *→* HRQOL); *SRMR = 0.000 CD = 0.179*						
Child abuse	−2.15	0.08	−.18	−27.39 ***	−2.31	−2.00
PA	1.00	0.05	0.11	18.22 ***	0.89	1.11
Child abuse * PA	0.15	0.06	0.02	2.39 *	0.03	0.27
Model 3 (X3 * W *→* HRQOL); *SRMR = 0.000 CD = 0.177*						
Household dysfunction	−1.14	0.06	−0.18	−25.81 ***	−1.55	−1.33
PA	0.98	0.05	0.11	18.52 ***	0.88	1.08
Household dysfunction * PA	0.03	0.04	0.004	0.61	−0.06	0.11
Model 4 (X1 * W *→* Physically Unhealthy Days); *SRMR = 0.000 CD = 0.162*						
ACEs	0.49	0.03	0.11	16.12 ***	0.43	0.55
PA	−0.98	0.04	−0.14	−23.18 ***	−1.06	−0.90
ACEs * PA	−0.04	0.03	−0.01	−1.70	−0.09	0.01
Model 5 (X2 * W *→* Physically Unhealthy Days); *SRMR = 0.000 CD = 0.162*						
Child abuse	0.99	0.06	0.10	16.23 ***	0.87	1.11
PA	−0.99	0.04	−0.14	−23.17 ***	−1.07	−0.90
Child abuse * PA	−0.13	0.05	−0.02	−2.51 *	−.23	−0.03
Model 6 (X3 * W *→* Physically Unhealthy Days); *SRMR = 0.000 CD = 0.159*						
Household dysfunction	0.61	0.04	0.09	13.90 ***	0.52	0.69
PA	−0.98	0.04	−0.14	−23.48 ***	−1.06	−0.89
Household dysfunction * PA	−0.04	0.03	−0.01	−1.01	−0.11	0.03
Model 7 (X1 * W *→* Mentally Unhealthy Days); *SRMR = 0.000 CD = 0.147*						
ACEs	0.97	0.03	0.22	29.38 ***	0.91	1.04
PA	−0.36	0.05	−0.05	−7.77 ***	−0.45	−0.27
ACEs * PA	−0.09	0.03	−0.02	−3.35 **	−0.14	−0.03
Model 8 (X2 * W *→* Mentally Unhealthy Days); *SRMR = 0.000 CD = 0.136*						
Child abuse	1.83	0.07	0.19	25.54 ***	1.69	1.97
PA	−0.39	0.05	−0.05	−8.16 ***	−0.48	−0.30
Child abuse * PA	−0.21	0.06	−0.03	−3.70 ***	−0.32	−0.10
Model 9 (X3 * W *→* Mentally Unhealthy Days); *SRMR = 0.000 CD = 0.137*						
Household dysfunction	1.27	0.05	0.19	25.80 ***	1.17	1.37
PA	−0.36	0.04	−0.05	−8.06 ***	−0.45	−0.27
Household dysfunction*PA	−0.13	0.04	−0.02	−3.39 **	−0.21	−0.05

*Note*. CI = Bootstrapping bias-corrected confidence interval. Table presents both standardized (β) and unstandardized (*B*) regression coefficients. Results of covariates were omitted. * *p* < 0.05, ** *p* < 0.01, and *** *p* < 0.001.

## Data Availability

The original data can be obtained via the CDC website-https://www.cdc.gov/brfss/annual_data/annual_2019.html (accessed on 3 January 2022).

## Abbreviations

ACEs	Adverse childhood experiences
BRFSS	Behavioral Risk Factor Surveillance System
CDC	Center for Disease Control
CDM	Covariate-dependent missingness
HRQOL	Health-related quality of life
MCAR	Missing is completely at random
ML	Maximum likelihood
PA	Physical activity
SEM	Structural Equation Modeling
